# Difficulties in the Prognostic Study of Oral Leukoplakia: Standardisation Proposal of Follow-Up Parameters

**DOI:** 10.3389/froh.2021.614045

**Published:** 2021-02-05

**Authors:** Cristina Saldivia-Siracusa, Wilfredo Alejandro González-Arriagada

**Affiliations:** ^1^Patología y Diagnóstico Oral, Facultad de Odontología, Universidad de Valparaíso, Valparaíso, Chile; ^2^Centro de Investigación Interoperativo en Ciencias Odontológicas y Médicas (CIICOM), Universidad de Valparaíso, Valparaíso, Chile

**Keywords:** oral leukoplakia, prognosis, follow-up, standardisation, oral potential malignant disorders

## Abstract

Oral leukoplakia is the most prevalent potentially malignant disorder of the oral cavity. To evaluate its potential for malignancy, appropriate documentation of the biological parameters is crucial, allowing the patients' progression to be assessed. We hypothesized a lack of standardization in the parameters employed for the prognostic study of oral leukoplakia; our aims were to determine the different parameters used for follow-up according to definition, importance, and frequency of use, and to provide a standardization proposal of follow-up research. We made a scoping review to identify papers with the keywords “leukoplakia,” “oral,” and “follow-up” published until June 2019 in English, Spanish and Portuguese literature through an online search in PUBMED, SCIELO, and SCOPUS databases. In total, 514 articles were initially identified, and fifty-nine publications were selected, of which 37 were retrospective. The reports included a total of 18,660 patients between 13 and 98 years old, with a mean age of 57.6 years. Tobacco and alcohol habits were positive for 77 and 37% of the patients, respectively. Our results showed that reported leukoplakias were predominantly located on buccal mucosa (40.4%), were homogeneous (60.8%), multiple (59.9%), smaller than 2 cm (74.4%) and histopathologically non-dysplastic (71%). The mean follow-up time was 55 months, with a 13% malignant transformation rate. The categorization and definition of multiple variables were notably diverse. Age, sex, habits (tobacco and alcohol), site, size, distribution, morphology, degree of dysplasia, and evolution were the chosen parameters for our proposal. The current study reflected the lack of consensus found in the literature regarding parameters for diagnosis or follow-up, impacting negatively on clinical and research results. standardization comprises an efficient way to facilitate the prognosis assessment of oral leukoplakia, being beneficial for clinical practice, and enabling better quality information to apply in research.

## Introduction

Oral and oropharyngeal squamous cell carcinomas is the most common of head and neck neoplasms, representing ~90% of malignancies in this region [1]. According to the 2018 IARC GLOBOCAN database, lip and oral cavity cancers represent 2.9% of all cancers in males and 1.0% in females [2]. They have a high morbidity and 5-year survival rate of <50%, which is strongly associated with late diagnosis. Therefore, early detection is a priority to reduce lethality.

Squamous cell carcinoma (SCC) has been directly related to the development of potentially malignant disorders, which are a group of lesions that suppose a risk of oral cancer development [3]. Oral leukoplakia (OL) is described as a white plaque of questionable risk, having excluded (other) known diseases or disorders that carry no increased risk of cancer [4]. The prevalence of oral leukoplakia is 2–3% [3], which increases after the third decade of life, with variations in sex predilection according to demographics [5]. Although its etiology is not completely elucidated, it has been linked to tobacco and alcohol consumption, which are often associated with the presentation of many leukoplakias [6]. However, lesions also occur in non-smoking and non-drinking patients, without any apparent etiology; these are known as idiopathic leukoplakias [3].

The malignant transformation rate of OL is between 1 and 2%, according to the WHO [3], with values ranging from 0.1 to 36.4% [7]. Hence, assessing the risk of potential malignant transformation of oral leukoplakia has been a major challenge, where clinical, histopathological, and molecular prognostic factors have been investigated. One of the contributing reasons for the incomplete understanding of the behavior of oral leukoplakia over time is the absence of a unifying recompilation method that allows a complete, objective, and specific patient information record to determine possible important factors to be considered prospectively regarding potential malignant transformation.

This investigation aims to use a scoping review to analyse the follow-up parameters currently used in the literature, and to provide a proposal for their standardization, to be applied for clinical use as a tool for the prognostic determination of patients with oral leukoplakia. For this purpose, our research question was: is there standardization in the follow-up parameters of oral leukoplakia for its prognostic study? Our main objective was to determine the different parameters used for follow-up and prognostic studies of oral leukoplakia, defining the most important ones, according to their importance and frequency of use. Finally, we propose a standardization of follow-up parameters for oral leukoplakia.

## Methods

### Study Design

A scoping review of the literature was carried out based on the criteria proposed by the PRISMA-ScR guide, to collect and classify the parameters used for the prognostic study of oral leukoplakia concerning its characteristics. Subsequently, a database with the selected articles was built and a descriptive analysis was performed.

### Eligibility Criteria

The inclusion criteria were (a) longitudinal studies of oral leukoplakia (including retrospective and prospective cohort studies, and experimental clinical trials), with a description of follow-up parameters; and (b) publications written in English, Spanish or Portuguese.

The exclusion criteria were (a) publications unrelated to the topic of the review; (b) a follow-up period of <6 months; (c) articles in which leukoplakia was grouped with other intraoral lesions considered or not as potentially malignant lesions; (d) animal trials; (e) extraoral lesions; (f) duplicates or double publications (keeping only the most recent one); and (g) papers that do not match the inclusion criteria and articles without full-text availability.

### Sources of Information

The electronic databases PubMed, SCOPUS, and SCIELO (June 2019) were selected to identify articles of potential relevance to this study. The search and selection of the articles was carried out by the authors.

### Search Strategy

The following keywords were used for the title or abstract: “oral leukoplakia” related to the term “follow-up” using the “AND” operator. In the Spanish and Portuguese languages, the keywords were “leucoplasia oral” and “seguimiento,” and “leucoplasia oral” and “acompanhamento,” respectively.

### Selection of Sources of Evidence

After the initial search, the articles were arranged in a basic documentary matrix, to eliminate duplicates and screen publications according to their relevance by title and summary. The remaining publications were then evaluated according to the inclusion and exclusion criteria, through screening and eligibility steps, creating a bibliometric matrix designed specifically for this study.

### Database

The selected articles were included in a database with the following data: title, author, country, publication date, objective, study design, inclusion and exclusion criteria, intervention, number of patients, number of leukoplakias, age (range), age (mean), sex, male:female ratio, race, alcohol habits, smoking, location, clinical appearance, degree of dysplasia, size, evolution, follow-up time, malignant transformation percentage, age of transformation (mean), estimated transformation time, mortality rate, immunohistochemistry, diet habits, and complementary parameters.

## Results

**Figure 1:**
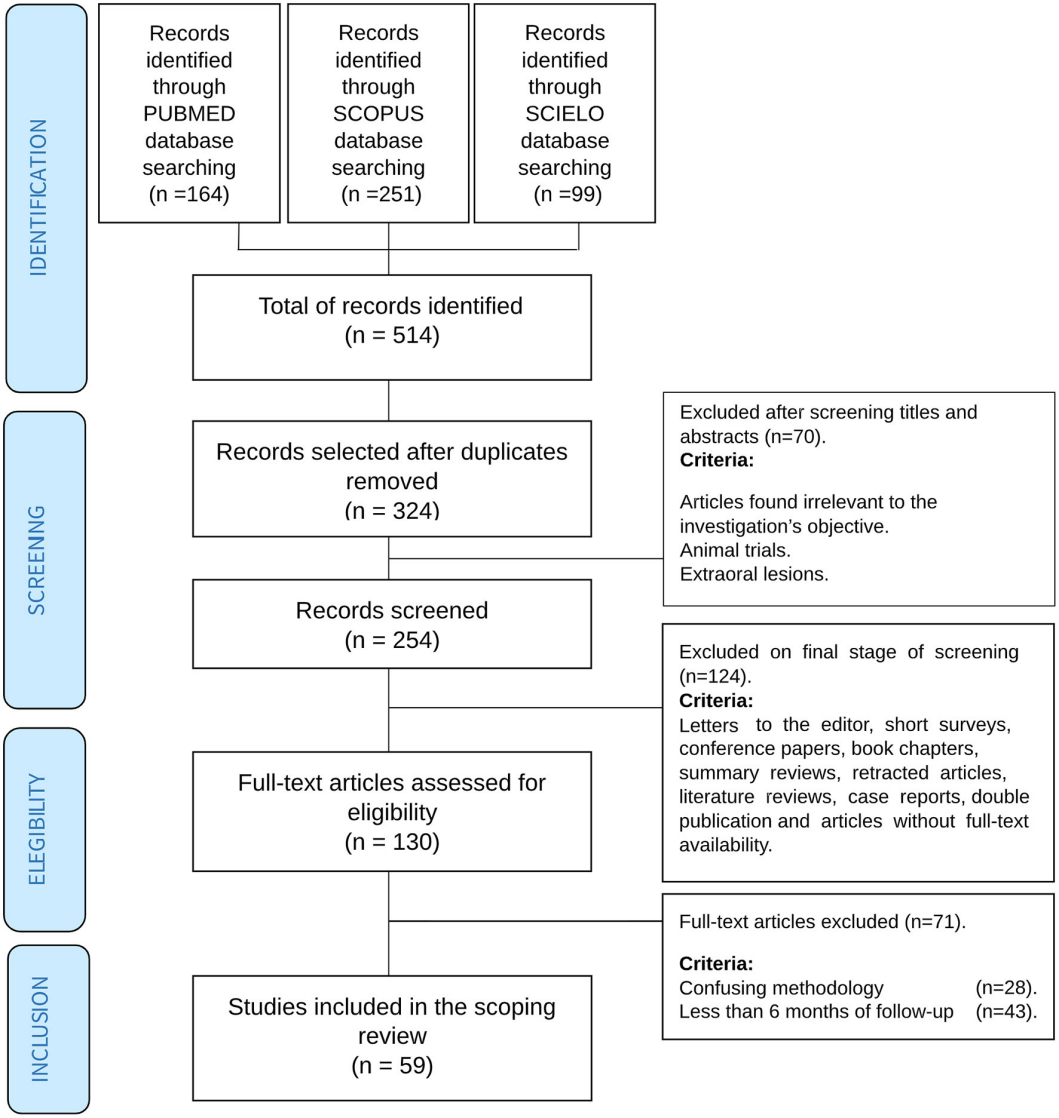
Selection process flowchart.

**Figure 2:**
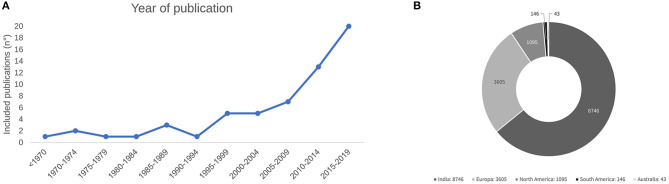
**(A)** Year of publication of the included publications. **(B)** Distribution of patients by region of origin.

The study selection process is presented, resulting in the selection of 59 articles [8–66] (Figure 1). Most publication dates range from 2015 to 2019 (Figure 2A), and were from Europe (22 publications), followed by Eastern Asia. The largest reported population was from India [39, 54] (Figure 2B).

A general database reflecting the distribution of the follow-up parameters was created to summarize each study. The frequency of the follow-up parameters and a summary of the results from the main clinicopathological parameters of selected articles is shown in Table 1. The most frequently used parameters found in our bibliometric matrix were chosen for this table. As seen, the most commonly used follow-up parameters were sex (58 studies), follow-up time (57 studies), malignant transformation percentage (57 studies), localisation (49 studies), age (46 studies), tobacco (43 studies), and degree of dysplasia (42 studies). Characteristics like alcohol (31 studies), clinical appearance (24 studies), distribution (22 studies), and size (20 studies) were less frequently reported. Other parameters were underreported, and therefore, not considered. It is important to highlight that no follow-up parameters were used in the studies that were finally selected.

**Table 1:** Clinicopathological features of the population of the articles included in the current study (summary of results).

Number of patients (59 studies)	18,660
Study design (59 studies)	Retrospective: 37 Prospective: 22
Intervention	Yes: 38 No: 21
Sex (male/female) (58 studies)	Male: 14,673 (79%) Female: 3,967 (21%)
Age (mean) (46 studies)	57.6 years
Age (range) (43 studies)	13–98 years
Tobacco (43 studies)	No: 33,323 (77%) Yes: 9,916 (23%)
Alcohol (31 studies)	No: 2,503 (63%) Yes: 1,457 (37%)
Localization (49 studies)	Buccal mucosa: 5,572 (40.44%) Tongue: 3,145 (22.82%) Gingiva: 1,577 (11.44%) Lip: 854 (6.2%) Palate: 835 (6.06%) Floor of mouth: 756 (5.49%) Alveolar mucosa: 489 (3.55%) Labial Commissures: 248 (1.80%) Retromolar trigone: 10 (0.07%) Other sites: 293 (2.13%)
Clinical appearance (24 studies)	Homogeneous: 2,278 (60.8%) Non-homogenous: 1,098 (29.3%) Verrucous: 371 (9.9%)
Distribution (22 studies)	Solitary: 565 (40.1%) Multiple: 844 (59.9%)
Size (20 studies)	<2 cm: 1,062 (74.4%) 2-4 cm: 236 (16.5%) >4 cm: 129 (9%)
Degree of dysplasia (trinary) (42 studies)	Non-dysplastic: 4,580 (71%) Mild dysplasia: 1,005 (15.6%) Moderate dysplasia: 637 (9.8%) Severe dysplasia: 297 (4.6%)
Degree of dysplasia (binary) (5 studies)	High grade: 220 (24.6%) Low grade: 673 (75.3%)
Evolution (34 studies)	No change: 3,124 (46.2%) Partial improvement: 1,739 (25.7%) Total improvement: 703 (10.4%) Progression: 590 (8.7%) Recurrence: 359 (5.3%) New lesions in other sites: 249 (3.7%)
Follow-up time (mean) (57 studies)	55 months
Follow-up time (range) (39 studies)	1-468 months
Malignant transformation percentage (mean) (57 studies)	13.07%

Concerning study design, 37 of the publications were retrospective and 22 prospective, and 38 of them included some type of intervention (Supplementary Table 1).

Regarding the collected data, a total of 18,660 patients were studied and followed over time, with 79% of them being male and 21% female, with an age range of 13-98 years (reported on 43 studies) and a mean age of 57.6 years (reported on 46 studies). With regard to habits, 43 articles stated tobacco use and 31 stated alcohol use, amounting to 77% of patients being tobacco-users, and 37% being alcohol-drinkers. The localisation of leukoplakia was reported in 49 studies, with buccal mucosa as the most frequent site (40.4%), followed by the tongue (22.8%). Labial commissures (1.8%) and retromolar trigone (0.07%) were the less frequently affected sites, and other non-specified sites represented 2.13% of cases. Clinical appearance was homogeneous in 60.8%, multiple (59.9%), and smaller than 2 cm (74.4%). Dysplasia was reported in 47 articles (42 with OMS-grading), and predominantly non-dysplastic (71%) or low grade (75.3% were low grade in 5 studies with a binary scale).

In those studies that stated evolution over time (34 studies), most of the leukoplakias did not change (46.2%), 5.3% recurred and 3.7% new lesions appeared.

The follow-up time ranged between 1 and 468 months (39 studies), with a mean of 55 months, and a malignant transformation percentage of 13%.

## Discussion

The current review identified 59 articles with a proper follow-up of oral leukoplakias from 1969 to 2019, with an increasing number in the last decade. The predominance of European and Asian publications stands out, with only three articles from Latin America (*n* = 146), corresponding to 0.78% of the total of reported patients. Currently, in Latin America, many of the therapeutic decisions are based on studies carried out in Europe, Asia, or North America, which may result in variations in data and therapy outcomes due to the regional impact of demographic and social characteristics. Considering the current results, we confirm the absence of standardization of the follow-up parameters of oral leukoplakias. It is crucial to emphasize that the high heterogeneity of parameters can be influenced by multiple methodological biases, such as selection bias (randomisation, inclusion, and exclusion criteria), information bias (calibration, masking), and analysis bias (variable selection, statistical analysis). A high disparity of recorded variables is observed, and some variables that we initially considered relevant turned out to be poorly reported, such as time of evolution and number of leukoplakias per patient.

Although the inclusion and exclusion criteria of the studies are not clinical follow-up parameters, their determination is imperative for conducting any study. Large differences in patient recruitment and clinical considerations for OL were observed, including articles that did not mention inclusion and exclusion criteria. Histological confirmation was the most important criterion, explicitly considered in 36 articles, although presenting multiple subjectivities when applied, like considering leukoplakias only with specific degrees of dysplasia [10, 14, 26, 62], or including non-dysplastic lesions [8, 19].

Regarding age, our results agree with the literature, with most affected patients being over 50 years-old [18, 45]. It has been estimated that <1% of affected men are under 30 years of age, and the risk of presentation increases in patients aged over 70 [57]. Additionally, we observed that oral potentially malignant disorders (OPMD) are more common in men [57], and some articles included in this study have a large male population [39, 54]. However, it is important to consider proliferative verrucous leukoplakia, which considers non-smoking elderly women as at risk patients, and contrasts with the classic profile [22, 67]. The number of female smokers in some countries has increased [68], which also has an impact on incidence distribution of OL. Even though the incidence of OL is higher in men, several studies mention superior malignant transformation in women, for unclear reasons [69].

The number of studies that do not track alcohol and tobacco use is striking, considering that these are already malignant transformation-associated factors supported by scientific evidence, so tracking habits or their cessation should be considered a priority. Discrepancies regarding the definition, quantity, and frequency of consumption were observed, both with smoking and alcohol [17, 50]. The multiplicity of criteria implies a wide variability in its categorisation. Regardless of the association of smoking with the presentation of oral leukoplakia, idiopathic lesions have been reported as having higher rates of malignancy [22, 68, 69]. While these data may be paradoxical, the cessation of tobacco and alcohol consumption should be advised to OL patients [70]. Finally, we consider it more convenient to categorize ex-smokers and ex-drinkers within the positive habit group than within the group that has never smoked or consumed alcohol.

Since there is a certain preponderance of patients of oriental origin, the use of areca or betel nut is worth mentioning, as it would be a highly influential risk factor of OL [71]. However, not enough data were collected based on the included studies, but their standardized recording is expected to be beneficial for future prognostic studies.

Location is a parameter that is generally reported, and the buccal mucosa is the most common location, followed by the tongue and gingiva [70]. In oriental studies, the localisation of OL may be influenced by cultural factors, as chewable tobacco or betel nut increases the risk of OL in less prevalent oral sites for western population [71, 72]. A combination of several regions of the oral cavity into a single group to categorize location is seen in various studies and confers a difficulty with assessing prognostic data in large investigations [27, 46]. Documenting the location of every lesion is very important, as is a detailed evaluation of the entire oral mucosa.

**Figure 3:**
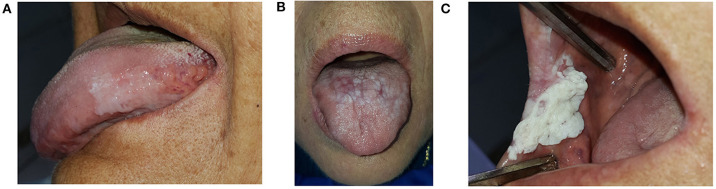
Clinical morphology of OL. **(A)** Homogeneous leukoplakia. **(B)** Non-homogeneous leukoplakia. **(C)** Verrucous leukoplakia.

As previously stated by Speight, regardless of OL being a predominantly white lesion, its dynamic progression can result in texture or color variations over time [72]. Most of the included studies used the morphological classification system of the WHO when recording clinical appearance (homogeneous and non-homogeneous, these last one presenting with nodular, verrucous and/or red areas). Some studies show a different prognostic behavior for homogeneous and non-homogeneous leukoplakia [22, 30]. Verrucous leukoplakia was mostly considered a subtype of non-homogeneous leukoplakia [73], and frequently involves a classic presentation of proliferative verrucous leukoplakia, which has been described as having a high rate of malignant transformation [22]. However, many studies still fail to describe the appearance of transformed leukoplakias, so it remains unclear whether verrucous leukoplakia has a greater predisposition to malignancy than homogeneous leukoplakia or not. Since there is scarce information in the literature, it would be useful to have data to elucidate particularities of the clinical presentation, and a strict photographic record during each control is necessary. We consider clinical appearance to be an important parameter to describe in follow-up research of leukoplakias, and we suggest classifying clinical morphology into 3 categories (Figure 3), as defined below. When documenting a single mixed lesion, the classification should be considered according to the predominant morphology:

- *Homogeneous leukoplakia*: White, non-removable, slightly elevated, uniform-looking lesion, with or without fissures/cracks [74].
- *Non-homogeneous leukoplakia*: Predominantly white, non-detachable, mixed appearance lesion, with some irregular areas of erythematous, and a granular or nodular surface (white or red polypoid growths or excrescences) [4].
- *Verrucous leukoplakia*: Non-removable white lesion with an exophytic surface or corrugated appearance, and digitiform or warty projections [73].

The presentation of multiple lesions and the extent of a leukoplakia has been associated with higher rates of malignant transformation [49, 63, 69]. The number of lesions in the oral mucosa as a clinical parameter was underreported and was included in only 20 studies. We believe that documenting the size and site of involvement could help to select a better criterion with which to distinguish multifocal lesions and single extensive lesions. We propose recording distribution as single or multiple, subdividing each individual lesion into “focal or localized” if they cover one anatomical zone, and “multifocal” if their extension involves two or more zones, as suggested by Monteiro et al. [41].

The categorisation of dysplasia is the gold standard parameter associated with the malignant transformation potential of oral leukoplakia [3]. In our database, the largest group corresponded to non-dysplastic lesions, followed by mild dysplasia. The reason for most of the leukoplakias being non-dysplastic could be related to the clinical misdiagnosis of white plaques and not excluding all other lesions like frictional keratosis that can exhibit a similar appearance, thus categorizing reactive or traumatic lesions as oral leukoplakias. It is important to encourage meticulousness and acuteness when performing visual intraoral assessment, as these findings could be a modifying factor regarding some of the obtained results, such as malignant transformation or mortality.

Concerning its classification, high heterogeneity was observed, including studies with binary-scale [21, 34–37] and trinary-scale graduation. Some other variations in classification were noted [10, 11, 27, 51], but they were omitted from Table 1. These non-standardized histopathological concepts can lead to data collection problems. Although it is an extremely relevant parameter due to the relationship between dysplasia and malignancy, not all dysplastic lesions progress to cancer [50]. The subjective estimation of dysplasia is controversial, and it has been discussed for several years whether the WHO graduation or the binary scale is the most appropriate. It is necessary to increase the study of molecular pathways that could be useful as a better tool for graduation; multiple biomarkers have been analyzed and stratified according to their characteristics with the aim of accurately determining the risk of progression to cancer and malignant transformation predictability, independently or in combination [67]. However, there are still significant limitations in the ongoing studies, such as small sample sizes, a lack of demographic information, the absence of control groups and/or limited follow-up data [75], making it difficult to report positive or negative predictive values for those biomarkers. Meanwhile, this paper reaffirms the importance of documenting the degree of dysplasia periodically when evaluating leukoplakia during its follow-up. For this purpose, it is always recommended to use the WHO scale, contributing to the homogeneity in the information reported.

While there have been postulations about the association between OPDM and superinfection by candida, histopathological changes may be regarded as reactive in nature. To date, it has not been possible to show a true causal relationship between candida, epithelial dysplasia, and cancer [72]. As not enough data were gathered regarding this topic, it would be of future interest to better describe the clinical aspects and histopathological findings when following-up OL patients.

Diverse intervention methods were reported, mainly surgical and laser implementation. Although there is a wide range of treatment options for oral leukoplakia, there is still no consensus on determining the most appropriate method to minimize its progression. It has been reported that advanced or treatment-resistant lesions may have a worse prognosis [23]. However, when comparing surgical excision with any other therapeutic method, there is no significant difference between treatment vs. no treatment in terms of malignant transformation rate [76]. We believe that standardizing the parameters for the follow-up of OLs in research studies will be tremendously useful for better evaluation of the results of interventional studies.

We define evolution as the course presented by patients since diagnosis, reported during the follow-up, including no changes in clinical presentation, total or partial improvements, progression in the extent or grade of dysplasia, malignant transformation, and new lesions in the oral cavity. This parameter was reported in 35 investigations, many with different definitions of recurrence [10, 17], reporting a recurrence rate of between 10 and 35% [57].

The follow-up period of most of the included studies was between 1 and 6 years. Only 4 studies continued for more than 9 years. According to Silverman [54], less than half of the patients with oral leukoplakia develop cancer in the first 2 years of diagnosis; other studies state that a longer follow-up time is associated with a higher number of malignant transformations [22, 74]. For these reasons, we can assume that the percentage of malignancy obtained (13%) would be higher with long-term surveillance, so conducting studies with a longer follow-up time is critical. There are no guidelines for the frequency and duration of OL follow-up, and some authors suggest lifelong monitoring, at intervals of 6 to 12 months [5]. Warnakulasuriya et al. recommend a more frequent follow-up in patients without intervention, approximately every 3 months [7], including patients without current risk factors, like patients with tobacco cessation [70]. Based on our review and to guarantee the validity of prognostic research in patients with oral leukoplakia, we propose that the follow-up should be at least 3 years long, checking in every 6 months and biopsy in case of clinical changes, suggestive of progression.

Malignant transformation rates are very variable. We observed a mean of 13.07%, ranging from 0.03 to 70.3%. These results may vary due to sample differences and length of follow-up period. Malignant transformation risk has been reported by up to 40% in non-smokers, high-risk locations, and those with a non-homogeneous appearance [73]. The mortality of oral SCC arising from OPMDs appears to be lower [50], but this discussion is still not certain, because we do not see every carcinoma in the initial phases.

It has been reported in the literature that the progression of oral leukoplakia is unpredictable [7], so patients with these lesions should be considered to have a potential risk of cancer, and hence there is a need for accurate research and scientific evidence. For this reason, a standardization of the follow-up parameters of oral leukoplakia patients is proposed below. The proposed parameters are summarized in Table 2. A simplified chart to be completed during clinical examination is shown in Table 3; with a second optional part with other parameters listed in Supplementary Table 2, according to the specific needs of care or research.

**Table 2:** Proposed parameters for standardization of follow-up of oral leukoplakias.

**Demographics:** Age (years and months). Sex (Male/female).
**Habits:** Tobacco • Presentation: combustible (cigarette, cigar, pipe, others) or non-combustible (smokeless: chewing, snoring, others). • Consumption time (years). • Consumption amount (per day). Areca nut • Consumption time (years). • Consumption amount (per day). Alcohol • Alcohol type: hard liquor, beer, or wine. • Consumption time (years). • Consumption amount (per day). The quantity of one (1) glass shall be considered as a measure. *Former habits will be registered as positive. Others (e.g., areca/betel nut): • Consumption time (years). • Consumption amount (per day). • Presentation: To be specified.
**Clinical:** • Distribution: solitary or multiple. • Solitary lesions may be subclassified as focal if encompassing a single anatomical site (e.g., hard palate), or multifocal if encompassing more than one anatomic area (e.g., extensive lesion involving lateral border of tongue and floor of mouth). • Site. • Size: Measurement - length x width (mm). • Clinical appearance: homogeneous, non-homogeneous o verrucous. *Mixed lesions will be categorized according to predominant morphology.
**Histopathology:** • Dysplasia: absence or presence. • Graduation scale according to WHO. *The degree of dysplasia should be recorded each time a new biopsy is performed, and it is recommended doing it so whenever clinical changes are seen.
**Evolution:** • New lesions (appears on different site- characteristics of every new lesion that appears after initial examination should also be documented). • No change (lesión remains clinically identical at follow-up). • Total improvement (lesion disappears). • Partial improvement (persistent lesión, with clinical changes [e.g., morphology, size, or biopsy-confirmed dysplasia]). • Progression (advance in size, clinical morphology, and/or degree of dysplasia confirmed by new biopsy). • Recurrence (new lesion that appears in the same site after it was removed).
**Follow-up time (in years and months)**
**Malignant transformation (if applicable):** To specify when and where (anatomic site), in case of multiple lesions.
**Observations:** To write any patient-specific notes.

**Table 3:**
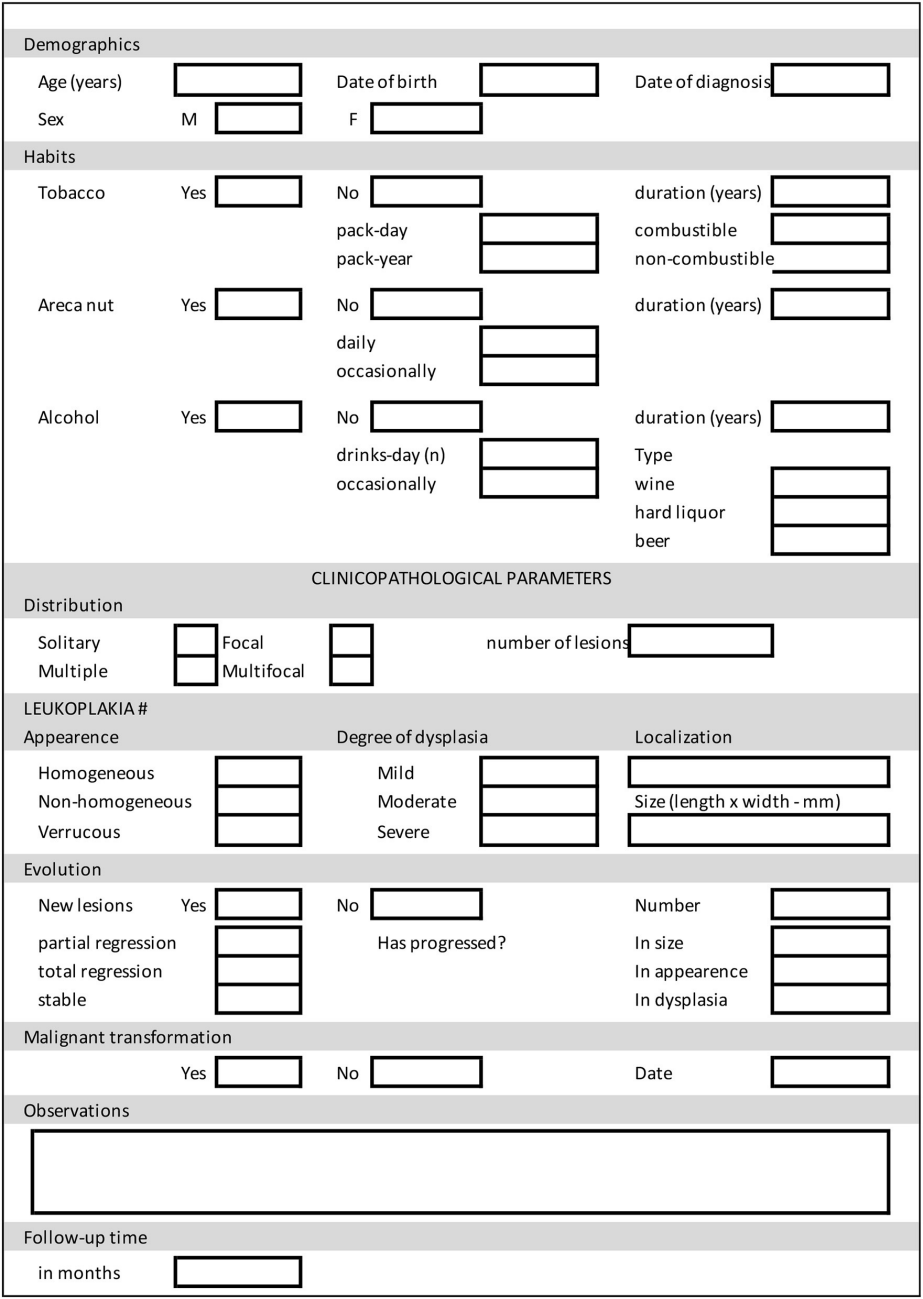
Datasheet for monitoring parameters for oral leukoplakia.

## Conclusions

The current review confirms the absence of standardization of the follow-up parameters of OL. The diversity of studies, the methodological differences, and the lack of uniformity of the parameters used for the evaluation of OL, not only lead to greater difficulty in obtaining accurate data for prognostic research purposes, but also hinder the treatment and monitoring of patients. A greater number of publications and longer follow-up periods will contribute to a better understanding and allow us to obtain new perspectives regarding the progression of OL, prognostic biomarkers and therapeutic options.

## Limitations

A particular limitation is noted given the nature of a scoping review and our set objective, which allowed us to select a wide amount of publications that show multiple disparities across their variables, making it difficult to summarize the research findings, leading to some biases that may have influenced our results. Our proposed standardization looks to minimize these complications in future prognostic research.

## Author Contributions

WG-A conceived the present idea. CS-S carried out the systematic selection of the articles under the supervision of WG-A. WG-A and CS-S verified the methodology and synthesized the results. Both authors discussed the results and contributed to the final manuscript.

## Conflict of Interest

The authors declare that the research was conducted in the absence of any commercial or financial relationships that could be construed as a potential conflict of interest.

## Supplementary Material

Supplementary Table 1 Bibliometric basis: Distribution of parameters used for oral leukoplakia follow-up.

Supplementary Table 2 Datasheet for monitoring parameters for oral leukoplakia: consideration of other parameters.
