# Supplementary material for: Difficulties in the Prognostic Study of Oral Leukoplakia: Standardisation Proposal of Follow-Up Parameters
Source: Front Oral Health. 2021 Feb 5;2:614045. doi: 10.3389/froh.2021.614045 (PMC8757698; doi:10.3389/froh.2021.614045)
Supplement: Supplementary Table 2 — Datasheet for monitoring parameters for oral leukoplakia: consideration of other parameters. [file Table_2.DOCX]

**Supplementary table II.** Datasheet for monitoring additional parameters for oral leukoplakia.
